# Immunological coagulation dual axis stratification identifies ultra high-risk phenotypes in systemic sclerosis

**DOI:** 10.3389/fimmu.2026.1866374

**Published:** 2026-07-02

**Authors:** Yaqi Zhao, Yan An, Qingrui Yang, Zhenzhen Ma

**Affiliations:** 1 Department of Rheumatology and Clinical Immunology, Peking Union Medical College Hospital, Chinese Academy of Medical Sciences, Peking Union Medical College, Beijing, China; 2 Department of Rheumatology and Immunology, Shandong Provincial Hospital, Cheeloo College of Medicine, Shandong University, Jinan, Shandong, China; 3 Department of Rheumatology and Immunology, Shandong Provincial Hospital Affiliated to Shandong First Medical University, Jinan, Shandong, China; 4 Department of Rheumatology and Immunology, Shengli Oilfield Central Hospital, Dongying, Shandong, China

**Keywords:** fibrinolysis, IICF network, immune-coagulation network, precision stratification, prognosis, systemic sclerosis

## Abstract

**Objective:**

Systemic sclerosis is a heterogeneous autoimmune disease characterized by complex interplay among immune activation, inflammation, coagulation, and fibrinolysis. This study aimed to construct a comprehensive immune-inflammation-coagulation-fibrinolysis (IICF) network and to evaluate its topological features and prognostic value across distinct organ involvement phenotypes.

**Methods:**

A total of 287 patients with systemic sclerosis were retrospectively enrolled, including 166 with isolated SSc, 85 with concomitant interstitial lung disease (ILD), 18 with pulmonary hypertension (PH), and 18 with both ILD and PH. Twelve composite indices encompassing IICF pathways were calculated. LASSO-Cox regression was employed to build a multi-index prognostic score, K-means clustering was performed to identify molecular endotypes, and patients were stratified using optimal cut-off values of the Systemic Immune-Inflammation Index (SII) and the D-dimer-to-Platelet Ratio (DPR) for dual-axis survival analysis.

**Results:**

Immune-coagulation dual-axis stratification revealed that patients with concurrent elevation of both SII and DPR had a 7.41-fold higher mortality risk than those with dual-low levels (HR 7.41, 95% CI 2.09–26.30, P = 0.002), whereas elevation of either index alone did not reach statistical significance. K-means clustering identified three IICF molecular endotypes, which showed incomplete concordance with the presence of ILD or PH. The multi-index prognostic score yielded an area under the curve of 0.747 for mortality prediction, surpassing the Prognostic Nutritional Index (0.693) and the Neutrophil-to-Lymphocyte Ratio (0.675).

**Conclusion:**

Adverse outcomes in systemic sclerosis are driven by systemic network disequilibrium rather than isolated pathway aberrations, with synergistic interplay between immune activation and coagulation dysregulation representing a pivotal mechanism. IICF-based dual-axis stratification and multi-index scoring provide a quantifiable approach for precision phenotyping and may inform stratified therapeutic decision-making.

## Introduction

Systemic sclerosis (SSc) is a prototypic multisystem connective tissue disease characterized by a complex interplay of aberrant immune activation, chronic inflammation, vascular endothelial injury, and dysregulated coagulation-fibrinolysis (1, 2). The clinical hallmarks of this disease are twofold. On the one hand, fibrosis of the skin and internal organs leads to altered organ structure and ultimately organ dysfunction. On the other hand, functional and structural vasculopathy manifests as Raynaud phenomenon, digital ulcers, pulmonary arterial hypertension, and renal crisis (3). In SSc, fibrosis and vasculopathy are intimately associated and lead to highly heterogeneous clinical manifestations with a widely variable prognosis (4). Traditional research has tended to analyse immune (5) or coagulation (6) markers in isolation; whilst this approach may aid in identifying specific biomarkers, it fundamentally overlooks the fact that SSc is a systemic network disease rather than a series of unrelated end-organ events.

Emerging evidence has delineated a sequential cascade driving SSc pathogenesis, which can be conceptualized as: Immune Activation-Inflammatory Storm-Endothelial Injury-Microthrombus Formation-Tissue Ischemia/Hypoxia-Fibrosis (7, 8). Within this intricate network, distinct biological modules fulfill specific, yet interdependent, roles. (1) Immune Cells as initiators: The innate and adaptive immune systems, including activated neutrophils, monocytes, and T/B cell-lymphocytes, serve as the primary igniters of the cascade, releasing profibrotic cytokines and directly damaging the microvasculature (9, 10). (2) Inflammatory Mediators as amplifiers: Acute-phase reactants act as potent amplifiers, propagating the inflammatory signal and modulating the acute-phase response that sustains endothelial activation (11, 12). (3) Coagulation System as effectors: The activation of the coagulation cascade—manifested by elevated fibrinogen, platelet hyperactivity, and increased D-dimer—functions as the effector limb, converting immunological and inflammatory stimuli into structural microvascular occlusive damage (13, 14). (4) Fibrinolytic Balance as repair regulators: The equilibrium of fibrinolysis, often reflected by the D-dimer/fibrinogen ratio, serves as a critical regulator of vascular patency and repair, dictating whether microthrombi resolve or progress to ischemic scarring (15, 16).

However, a single metric can only capture a single node within a network; it cannot reflect the interactions between nodes or the network’s topological characteristics. To address this knowledge gap, the present study employs an innovative multi-dimensional integration strategy to construct and validate a comprehensive Immune-Inflammation-Coagulation-Fibrinolysis (IICF) network. By moving beyond the analysis of isolated markers, we aim to elucidate the systemic network imbalance patterns specific to distinct SSc organ manifestations. We therefore hypothesize that the synergistic interplay between immune activation and coagulation dysregulation constitutes an independent risk factor for mortality in SSc. Specifically, we postulate that patients with concurrent elevation of both SII and DPR will exhibit a disproportionally higher mortality risk compared to those with single-axis or no elevation, and that this risk differential cannot be captured by single markers or conventional organ-based stratification. Deciphering this systemic network architecture may provide novel insights into precision phenotyping and risk-stratified management in this heterogeneous and challenging autoimmune disease.

## Materials and methods

### Study design and participants

This study enrolled 287 patients diagnosed with systemic sclerosis (SSc) during hospitalization at Shandong Provincial Hospital between 2013 and 2023. Each case fulfilled the 2013 ACR/EULAR classification criteria (17) for systemic sclerosis and was confirmed by at least two rheumatology specialists. According to the 2022 European Society of Cardiology (ESC)/European Respiratory Society (ERS) Guidelines for the Diagnosis and Treatment of Pulmonary Hypertension, pulmonary hypertension (PH) is now defined by a mean pulmonary arterial pressure >20 mmHg measured at rest by right heart catheterization (RHC) (18). Although RHC constitutes the clinical reference standard for PH, its invasive nature and attendant complications preclude routine application (19). Consequently, the present study employed echocardiography to identify indirect sonographic signs of PH. The echocardiographic screening threshold was a systolic pulmonary arterial pressure ≥36 mmHg (1 mmHg = 0.133 kPa). The diagnosis of interstitial lung disease (ILD) relied on consensus between radiologists and pulmonologists, based upon high-resolution computed tomography (HRCT) demonstrating bilateral reticular opacities, irrespective of concomitant traction bronchiectasis (20). In the context of limited or diffuse cutaneous systemic sclerosis, the occurrence of scleroderma renal crisis was established by the following criteria (21): Blood pressure criterion—sustained blood pressure >150/85 mmHg documented on at least two separate measurements within a 24−hour period; this pressure threshold qualifies as significant hypertension per New York Heart Association standards. Renal function criterion—documented decline in renal function, defined as a reduction of at least 30% in the estimated glomerular filtration rate. Where feasible, initial findings were corroborated by repeat measurement of serum creatinine concentration and recalculation of the glomerular filtration rate. Further support for the diagnosis of acute renal crisis was provided by any of the following ancillary manifestations, when present: (1) microangiopathic hemolytic anemia evident on peripheral blood smear; (2) characteristic hypertensive retinopathy of acute crisis; (3) newly detected urinary erythrocytes (in the absence of alternative aetiologies); (4) acute pulmonary edema; (5) oliguria or anuria; (6) renal biopsy revealing characteristic pathological changes; (7) if renal biopsy demonstrated an alternative etiology, the case was excluded from the classification of scleroderma renal crisis. Gastrointestinal involvement was defined by the presence of esophageal dysmotility, gastroparesis, small intestinal bacterial overgrowth, colonic dysmotility, or fecal incontinence. Cardiac involvement was defined as elevated N−terminal pro−B−type natriuretic peptide (NT−proBNP) accompanied by elevated cardiac troponin T and/or evidence of structural and/or functional impairment identified via echocardiography or cardiac magnetic resonance imaging (MRI) demonstrating left ventricular dysfunction, provided no alternative etiology more plausibly accounted for these findings (22). Cutaneous involvement was defined by the presence of digital swelling; skin thickening, induration, and tightness; digital pitting or flexion contractures; and salt−and−pepper skin changes (23). The study was conducted in accordance with the principles of the Declaration of Helsinki and received approval from the Ethics Committee of Shandong Provincial Hospital Affiliated to Shandong First Medical University (Ethics Approval No: 2022−413). Written informed consent was obtained from all participants.

### Detailed inclusion and exclusion criteria

#### Inclusion criteria

1. Fulfillment of the 2013 ACR/EULAR classification criteria for systemic sclerosis.
2. Age between 18 and 80 years with disease duration ≥6 months.
3. Availability of complete baseline data, including complete blood count with differential, coagulation profile ((including prothrombin time, activated partial thromboplastin time, fibrinogen, and D-dimer), hepatic and renal function tests, high−sensitivity C−reactive protein (hs−CRP), and autoantibody profile.
4. The study was approved by the Ethics Committee with a waiver of informed consent due to the retrospective design, and written informed consent was obtained from patients who were contactable during follow-up.

#### Exclusion criteria

1. Coexistence of other autoimmune diseases.
2. Acute infection, surgery, trauma, or active hemorrhage within 1 month prior to the index hospitalization/admission.
3. Use of anticoagulants or antiplatelet agents at baseline.
4. Severe hepatic dysfunction (Child-Pugh Class C) or severe renal insufficiency (serum creatinine >2.5 mg/dL or eGFR <30 mL/min/1.73 m^2^).
5. Active malignancy or malignancy diagnosed within the past 5 years, pregnancy at the time of enrollment, or hematologic disorders (e.g., immune thrombocytopenia, leukemia).
6. Recent (within 3 months) administration of intravenous cyclophosphamide or biologic agents.

### Data extraction

A retrospective review of medical records was conducted for all enrolled patients. Extracted demographic data encompassed sex, age, and disease duration. Clinical characteristics comprised skin thickening; gastrointestinal manifestations (esophageal symptoms including dysphagia and/or regurgitation, gastric symptoms including early satiety and/or vomiting, intestinal symptoms including diarrhea, bloating, and/or constipation); vascular involvement (Raynaud’s phenomenon, digital ulcers); joint contractures; fever; xerostomia; xerophthalmia; anemia; hematuria; proteinuria; and polyserositis. Laboratory investigations included complete blood count, biochemical panel, cardiac biomarkers (creatine kinase-MB and cardiac troponin T), NT−proBNP, and autoantibody profile (antinuclear antibody, anti−double−stranded DNA antibody, anti−SSA antibody, anti−SSB antibody, anti−U1 small nuclear ribonucleoprotein antibody, anti−Jo−1 antibody, anti−Scl−70 antibody, anti−cyclic citrullinated peptide antibody, rheumatoid factor, and immunoglobulins). Ancillary assessments comprised pulmonary function tests (diffusing capacity of the lung for carbon monoxide and forced vital capacity), echocardiography, and high−resolution computed tomography of the chest. Treatment data included previous or current use of glucocorticoids or immunosuppressive agents. Follow−up was performed via telephone to ascertain survival status, follow-up duration for living patients, time of death, and cause of death for deceased patients.

### Twelve composite indices

The following 12 indices were calculated: Neutrophil−to−Lymphocyte Ratio (NLR): Absolute neutrophil count divided by absolute lymphocyte count; Eosinophil−to−Lymphocyte Ratio (ELR): Absolute eosinophil count divided by absolute lymphocyte count; C−Reactive Protein−to−Albumin Ratio (CAR): C−reactive protein (mg/L) divided by albumin (g/L); Red Cell Distribution Width−to−Albumin Ratio (RAR): Red cell distribution width (fL) divided by albumin (g/L); Prognostic Nutritional Index (PNI): Calculated as albumin (g/L) + 5 × peripheral blood lymphocyte count (×10⁹/L); Systemic Immune−Inflammation Index (SII): Calculated as platelet count (×10⁹/L) × absolute neutrophil count (×10⁹/L)/absolute lymphocyte count (×10⁹/L); Inflammatory Burden Index (IBI): Calculated as C−reactive protein (mg/L) × absolute neutrophil count (×10⁹/L)/absolute lymphocyte count (×10⁹/L); Platelet−to−Lymphocyte Ratio (PLR): Absolute platelet count divided by absolute lymphocyte count; D−Dimer−to−Platelet Ratio (DPR): Calculated as D−dimer (mg/L) divided by platelet count (×10⁹/L); Fibrinogen−to−Albumin Ratio (FAR): Calculated as fibrinogen (g/L) divided by albumin (g/L); D−Dimer−to−Fibrinogen Ratio (DFR): Calculated as D−dimer (mg/L) divided by fibrinogen (g/L); C−Reactive Protein−to−D−Dimer Ratio (CRP_DD): Calculated as C−reactive protein (mg/L) divided by D−dimer (mg/L). These 12 composite indices were selected to systematically represent the four interconnected biological modules of the IICF network. NLR, ELR, SII, IBI, and PLR capture immune cell activation and the balance between innate and adaptive immunity. CAR, RAR, and FAR reflect the interplay between systemic inflammation and nutritional or metabolic status. PNI integrates nutritional reserve with immune competence. DPR, DFR, and CRP_DD capture the interface between coagulation activation, fibrinolytic turnover, and inflammatory burden. Together, these indices span the full IICF cascade from immune initiation through inflammatory amplification, coagulation effector function, and fibrinolytic regulation.

### Missing value handling and data preprocessing

To maximize data utilization, missing values for continuous variables were imputed via multiple imputation by chained equations (MICE) using the “mice” package. Prior to imputation, 19 non−core variables exhibiting high collinearity (Spearman |r| > 0.9) with other variables—including anti−U1RNP antibody, hematocrit, lymphocyte percentage, white blood cell count, and others—were identified and excluded. Subsequently, multiple imputation with a ridge penalty (ridge=0.1) was performed on the remaining dataset comprising 127 continuous variables, generating m=5 imputed datasets. The post−imputation dataset retained its original dimensions of 287 cases × 245 variables, thereby preserving sample integrity for subsequent analyses.

### Correlation network analysis and visualization

Spearman rank correlation coefficients were computed for all continuous variables and the 12 core immune-inflammation-coagulation-fibrinolysis (IICF) indices to generate a correlation matrix. Correlation heatmaps were constructed using the R package “pheatmap”. Correlation networks were built based on a threshold of |r| > 0.3 and P < 0.05, wherein nodes represent individual indicators and edges denote significant correlations. Network construction and visualization were accomplished using the “igraph” package.

### Construction and evaluation of prognostic prediction models

With patient prognosis (mortality) as the outcome variable, the discriminatory capacity of the 12 IICF indices was assessed via receiver operating characteristic (ROC) curve analysis. The area under the curve (AUC), optimal cut−off value, sensitivity, specificity, and Youden index were computed. Optimal cut−off values were determined by maximizing the Youden index. Comparisons of AUCs among different indices were performed using the DeLong test. To identify independent prognostic factors and develop a multi−index combined model, the 12 IICF indices were subjected to LASSO−Cox regression with 10−fold cross−validation to select the optimal penalty parameter λ. Variables with non−zero coefficients were retained for entry into a multivariable Cox proportional hazards regression model. Based on the six indices ultimately retained in the Cox model (NLR, ELR, CAR, RAR, DPR, and CRP_DD), a combined risk score (designated the IICF−6 Risk Score) was constructed via linear weighting of the respective regression coefficients. Model discrimination was evaluated using Harrell’s C−index, and survival differences between high− and low−risk groups were compared using Kaplan–Meier curves. The Cox regression model satisfied the proportional hazards assumption. Statistical significance was set at P < 0.05.

### Stratified analysis by immune–coagulation dual−axis

Patients were stratified into four quadrants based on the optimal cut−off values (derived from ROC analysis) of the immune−axis representative index SII and the coagulation−axis representative index DPR: Quadrant I (high SII + high DPR), Quadrant II (low SII + high DPR), Quadrant III (low SII + low DPR), and Quadrant IV (high SII + low DPR). Survival differences among the four groups were assessed using the Kaplan–Meier method. With Quadrant III serving as the reference, hazard ratios (HRs) and 95% confidence intervals for patient prognosis were calculated for each quadrant via univariate Cox regression.

### Dimensionality reduction visualization and unsupervised clustering

Following Z−score standardization of the 12 IICF indices, dimensionality reduction was performed using t−distributed stochastic neighbor embedding (t−SNE) and uniform manifold approximation and projection (UMAP). t−SNE parameters were set as follows: perplexity=30, number of iterations=1000. UMAP parameters were set as: n-neighbors=15, min-dist=0.1. Unsupervised classification of patients was carried out using K−means clustering. The optimal number of clusters (k) was determined comprehensively by the elbow method and the silhouette coefficient, with within−cluster sum of squares (WSS) and average silhouette width computed for k ranging from 2 to 10. The stability of the clustering solution was validated using hierarchical clustering (Ward’s method, Euclidean distance). The feature profiles of each cluster were visualized as a heatmap of Z−score means.

### Latent class analysis

To verify the robustness of the clustering structure, the 12 IICF indices were discretized into ordered categorical variables based on tertiles, and latent class analysis (LCA) was performed. Models specifying 1 to 6 latent classes were fitted, and the optimal number of classes was selected according to the minimum Bayesian information criterion (BIC). LCA classification results were visualized using t−SNE plots and cross−tabulated against clinically defined ILD/PH subgroups. LCA was implemented using the R package “poLCA”.

### Statistical analysis

Statistical analyses were conducted using IBM SPSS (version 26.0) and R software (version 4.2.1). Quantitative variables are presented as mean ± standard deviation (for normally or approximately normally distributed data) or as median and interquartile range (for non−normally distributed data). Categorical variables are expressed as frequencies and percentages. Depending on data distribution, intergroup comparisons were performed using one−way analysis of variance (ANOVA) or the Kruskal–Wallis test for continuous variables, and the chi−square test or Fisher’s exact test for categorical variables.

## Result

### Baseline characteristics of the study population and group stratification

A total of 287 patients with systemic sclerosis were enrolled in this study, comprising 166 cases of isolated SSc, 85 cases of SSc−ILD, 18 cases of SSc−PH, and 18 cases of SSc−ILD−PH. The overall cohort was predominantly female (230/287, 80.1%), with a mean age of 50.31 ± 14.27 years and a median disease duration of 30 (interquartile range, 12–84) months. Statistically significant differences were observed across the four groups with respect to age, disease duration, esophageal symptoms, Raynaud’s phenomenon, serositis, left ventricular ejection fraction (EF), NT−proBNP levels, pulmonary arterial pressure, diffusing capacity of the lung for carbon monoxide (DLCO) expressed as % predicted, proteinuria, autoantibody profiles (anti−U1 ribonucleoprotein antibody, antinuclear antibody, anti−histone antibody, anti−Scl70 antibody), as well as the composite inflammatory indices CAR, PLR, and FAR, and therapeutic regimens (all P < 0.05). Specifically, patients with coexisting ILD and/or PH were older and had longer disease duration compared with those with isolated SSc (P < 0.05). The prevalence of esophageal symptoms was higher in the SSc−ILD group (24.7%) than in the isolated SSc group (12.0%), whereas Raynaud’s phenomenon was least frequent in the SSc−PH group (44.4%), which was significantly lower than in the other groups (P < 0.001). Serositis was most prominent in the SSc−PH group (50.0%), in contrast to only 4.2% in the isolated SSc group (P < 0.001). Regarding cardiopulmonary parameters, pulmonary arterial pressure was markedly elevated in the two groups with PH (SSc−PH: 48.72 ± 14.24 mmHg; SSc−ILD−PH: 52.44 ± 16.95 mmHg), accompanied by reduced EF and substantially increased NT−proBNP concentrations (P < 0.05). DLCO (% predicted) was lowest in the SSc−ILD−PH group (39.56 ± 14.59%) and relatively preserved in the isolated SSc group (86.82 ± 28.34%) (P = 0.001). With respect to renal involvement, proteinuria was more common in the SSc−ILD (28.2%) and SSc−PH (33.3%) groups (P < 0.001). Autoantibody profiling revealed a high prevalence of anti−Scl−70 positivity in the SSc−ILD group (41.2%), significantly exceeding that of the other groups (P < 0.001), whereas AHA positivity was detected exclusively in the SSc−ILD group (3.5%). Among inflammation−related composite indices, CAR and FAR exhibited an upward trend in patients with ILD or PH, and PLR attained the highest median level in the SSc−ILD group (166.92, P = 0.003). Concerning treatment, mycophenolate mofetil was most frequently administered in the SSc−PH group (61.1%), whereas methotrexate was more commonly prescribed in the isolated SSc group (13.9%). During a median follow-up of 108 (IQR 98–117) months, the isolated SSc group had the highest survival rate (98.2%), whereas the SSc-PH group had the lowest (66.7%), with a statistically significant difference among the groups (P < 0.001, log-rank test). Detailed data are presented in **Table 1**.

**Table 1:** Characteristics of the SSc patients in the 4 subgroups.

Variables	All patients(n=287)	SSc (n=166)	SSc-ILD(n=85)	SSc-PH(n=18)	SSc-ILD-PH(n=18)	*p*
Demographic characteristics						
Gender(M/F)	57/230	40/126	12/73	3/15	2/16	0.057
Age(years)	50.31 ± 14.27	51.00 ± 15.08	54.00 ± 12.72	53.00 ± 13.75	54.00 ± 8.81	0.005*
Disease duration(months)	30(12,84)	24(10,72)	48(12,120)	12(7,72)	54(21,87)	0.041*
SSc characteristics						
Cutaneous involvement (n,%)	273(95.1%)	159(95.7%)	81(95.2%)	17(94.4%)	16(88.9%)	0.566
Esophageal symptoms(n,%)	47(16.4%)	20(12.0%)	21(24.7%)	5(27.8%)	1(5.6%)	0.007*
Stomach symptoms(n,%)	78(27.2%)	42(25.3%)	28(32.9%)	5(27.8%)	3(16.7%)	0.349
Intestinal symptoms(n,%)	9(3.1%)	4(2.4%)	3(3.5%)	1(5.56%)	1(5.56%)	0.068
Joint contractures	127(44.3%)	75(45.2%)	36(42.4%)	8(44.4%)	8(44.4%)	0.970
Raynaud's symptoms(n,%)	239(90.2%)	142(85.5%)	73(85.9%)	8(44.4%)	16(88.9%)	0.000***
Finger ulcers(n,%)	26(9.1%)	14(8.4%)	8(9.4%)	1(5.6%)	3(16.7%)	0.555
Serositis(n,%)	31(10.8%)	7(4.2%)	13(15.3%)	9(50.0%)	2(11.1%)	0.000***
EF(median,%)	62.00(61.77,63.00)	62.00(61.80,63.00)	62.00(61.78,63.00)	61.00(60.00,62.00)	61.90(60.00,62.25)	0.001*
NT-pro-BNP(pg/ml)	207.10(93.20,732.18)	170.00(83.80,377.90)	177.90(75.91,456.67)	2183.00(392.15,6605.50)	268.35(132.93,3943.93)	0.023*
Pulmonary artery pressure (mmHg)	32.17 ± 9.76	29.55 ± 4.72	29.47 ± 3.71	48.72 ± 14.24	52.44 ± 16.95	0.000***
DLCO actual/forecast(%)	59.09 ± 22.25	86.82 ± 28.34	58.30 ± 17.41	–	39.56 ± 14.59	0.001*
FVC(%)	74.13 ± 20.88	84.00 ± 22.88	76.72 ± 20.00	–	56.26 ± 14.85	0.051
Scleroderma renal crisis (n,%)	13(4.5%)	4(2.4%)	5(5.9%)	2(11.1%)	2(11.1%)	0.054
Proteinuria(n,%)	37(12.9%)	4(2.4%)	24(28.2%)	6(33.3%)	3(16.7%)	0.000***
Hypertension(n,%)	42(14.6%)	24(14.5%)	13(15.3%)	3(16.7%)	2(11.1%)	0.955
U1RNP positive(n,%)	27(9.4%)	10(6.0%)	11(12.9%)	2(11.1%)	4(22.2%)	0.038*
ANA positive(n,%)	265(92.3%)	148(89.2%)	81(95.3%)	18(100.0%)	18(100.0%)	0.031*
AHA positive(n,%)	3(1.0%)	0(0.0%)	3(3.5%)	0(0.0%)	0(0.0%)	0.035*
SCL-70 positive(n,%)	74(25.8%)	30(18.1%)	35(41.2%)	3(16.7%)	6(33.3%)	0.000***
ACA positive(n,%)	66(23.0%)	45(27.1%)	13(15.3%)	4(22.2%)	4(22.2%)	0.150
RF positive(n,%)	44(15.3%)	18(10.8%)	18(21.2%)	3(16.7%)	5(27.8%)	0.052
NLR	2.40(1.79,3.50)	2.33(1.57,3.12)	2.51(1.96,4.29)	3.17(1.88,5.44)	2.70(2.03,3.18)	0.483
ELR	0.04(0.02,0.09)	0.04(0.02,0.08)	0.03(0.01,0.10)	0.01(0.00,0.09)	0.03(0.00,0.07)	0.842
CAR	0.08(0.02,0.15)	0.07(0.01,0.10)	0.08(0.02,0.41)	0.12(0.01,0.41)	0.17(0.04,0.28)	0.008*
RAR	2.21(1.75,2.76)	2.20(1.73,2.78)	2.19(1.78,2.64)	2.18(1.63,3.19)	2.21(1.73,2.70)	0.860
PNI	47.70(43.00,51.98)	49.05(45.30,52.50)	45.00(40.70,50.30)	45.15(40.67,51.40)	45.70(39.90,48.93)	0.727
SII	552.79(368.28,891.03)	516.31(343.16,770.12)	634.74(419.23,1221.70)	623.06(381.83,1247.30)	451.93(362.71,750.23)	0.240
IBI	6.45(1.78,15.11)	4.34(1.66,11.48)	7.70(1.77,38.48)	9.96(2.03,53.23)	12.65(4.68,26.09)	0.254
PLR	145.18(108.58,199.62)	134.10(101.77,178.55)	166.92(118.97,234.06)	139.94(102.74,188.00)	134.64(106.43,169.66)	0.003*
DPR	0.001(0.000,0.004)	0.001(0.000,0.002)	0.001(0.001,0.004)	0.004(0.000,0.012)	0.004(0.001,0.0108)	0.204
FAR	0.07(0.06,0.09)	0.07(0.06,0.08)	0.07(0.06,0.11)	0.07(0.05,0.09)	0.08(0.05,0.11)	0.024*
DFR	0.31 ± 0.52	0.21 ± 0.33	0.38 ± 0.67	0.50 ± 0.64	0.50 ± 0.71	0.086
CRP_DD	5.16(1.42,17.35)	7.18(1.49,17.31)	3.58(0.93,15.03)	6.91(2.62,55.02)	10.32(1.32,29.00)	0.052
Treatment						
Past or current steroids(n,%)	259(90.2%)	145(87.3%)	79(92.9%)	17(94.4%)	18(100.0%)	0.052
Past or current Cyclophosphamide(n,%)	6(2.1%)	4(2.4%)	2(2.4%)	0(0.0%)	0(0.0%)	0.744
Past or current Mycophenolate Mofetil (n,%)	122(42.5%)	59(35.5%)	44(51.8%)	11(61.1%)	8(44.4%)	0.024*
Past or current Methotrexate(n,%)	28(9.8%)	23(13.9%)	5(5.9%)	0(0.0%)	0(0.0%)	0.008*
Prognosis						
Survivor(n,%)	261(90.9%)	163(98.2%)	71(83.5%)	12(66.7%)	15(83.3%)	0.000***

Esophageal symptoms included dysphagia and/or reflux, stomach symptoms included early satiety and/or vomiting, EF, Ejection fraction; NT-pro-BNP, N-terminal pro-brain natriuretic peptide; DLCO actual/forecast, Diffusing capacity of the lung for carbon monoxide % predicted; FVC, Forced vital capacity; U1RNP positive, Anti-U1 ribonucleoprotein antibody; ANA positive, Antinuclear antibody; AHA positive, Anti-histone antibody; SCL-70 positive, Anti-Scl-70 antibody; ACA positive, Anti-centromere antibody; RF positive, Rheumatoid factor; NLR, Neutrophil-to-lymphocyte ratio; ELR, Eosinophil-to-lymphocyte ratio; CAR, C-reactive protein-to-albumin ratio; RAR, Red cell distribution width-to-albumin ratio; PNI, Prognostic nutritional index; SII, Systemic immune-inflammation index; IBI, Inflammatory burden index; PLR, Platelet-to-lymphocyte ratio; DPR, D-dimer-to-platelet ratio; FAR, Fibrinogen-to-albumin ratio; DFR, D-dimer-to-fibrinogen ratio; CRP_DD, C-reactive protein-to-D-dimer ratio. Characteristics were compared among groups using one-way ANOVA or Mann-Whitney U test as appropriate. P < 0.05, P < 0.001.

### Intercorrelations among IICF indices and clinical variables

The present study focused on 12 composite indices reflecting integrated IICF pathways, namely NLR, ELR, CAR, RAR, PNI, SII, IBI, PLR, DPR, FAR, DFR, and the CRP_DD. Spearman correlation analysis performed on all continuous variables and visualized as a heatmap (**Supplementary Material 1**) revealed an extensive network of associations across functionally distinct categories of variables, underscoring the interplay among markers of different physiological systems.

### Correlation network of the 12 indices in the overall cohort and stratified by subgroup

**Figure 1:**
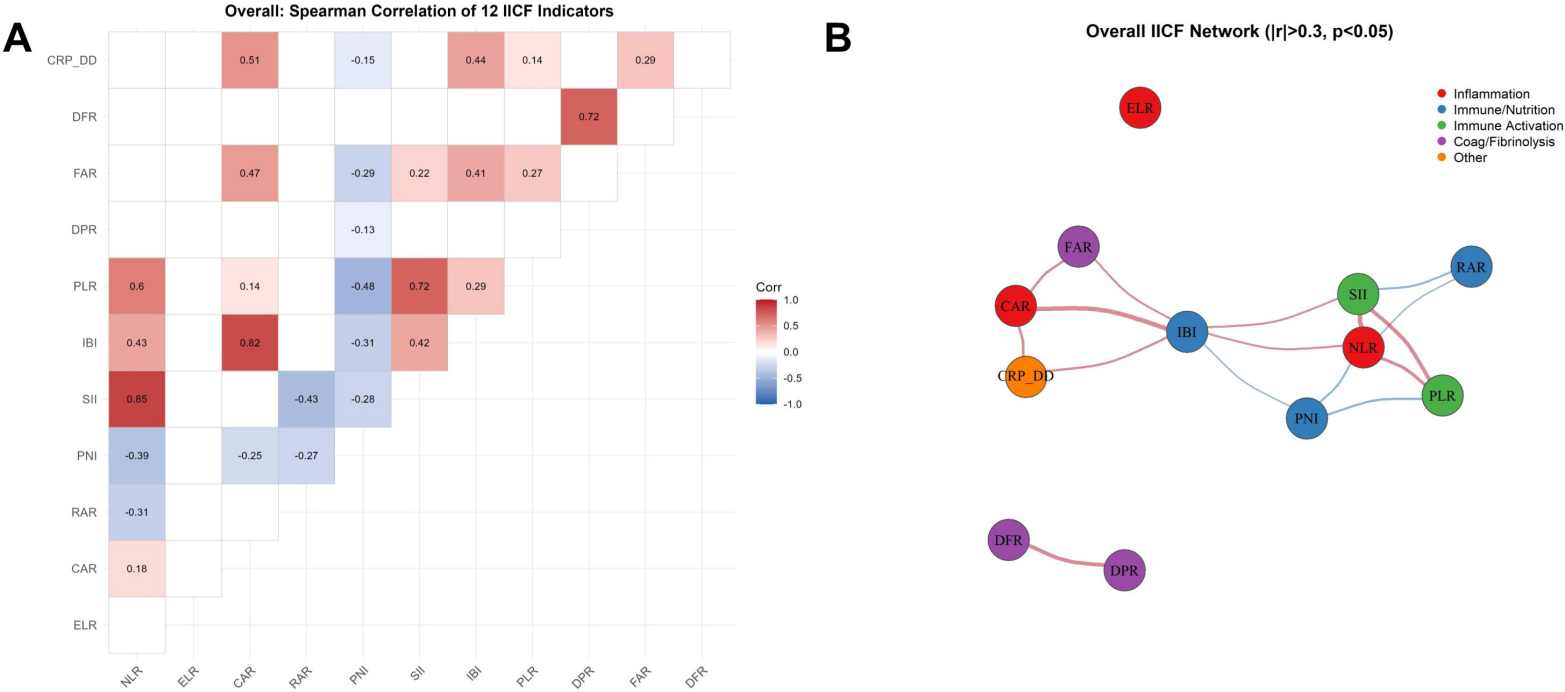
Overall spearman correlation and network analysis of 12 IICF indicators. **(A)** Spearman correlation heatmap of 12 integrated inflammation-coagulation-fibrinolysis (IICF) indicators in the total cohort (n=287). **(B)** Correlation network constructed with the threshold of |r| > 0.3 and P < 0.05. Nodes represent individual indicators, edges represent statistically significant correlations, and different node colors indicate distinct functional modules (inflammation, immune/nutrition, immune activation, coagulation/fibrinolysis).

**Figure 2:**
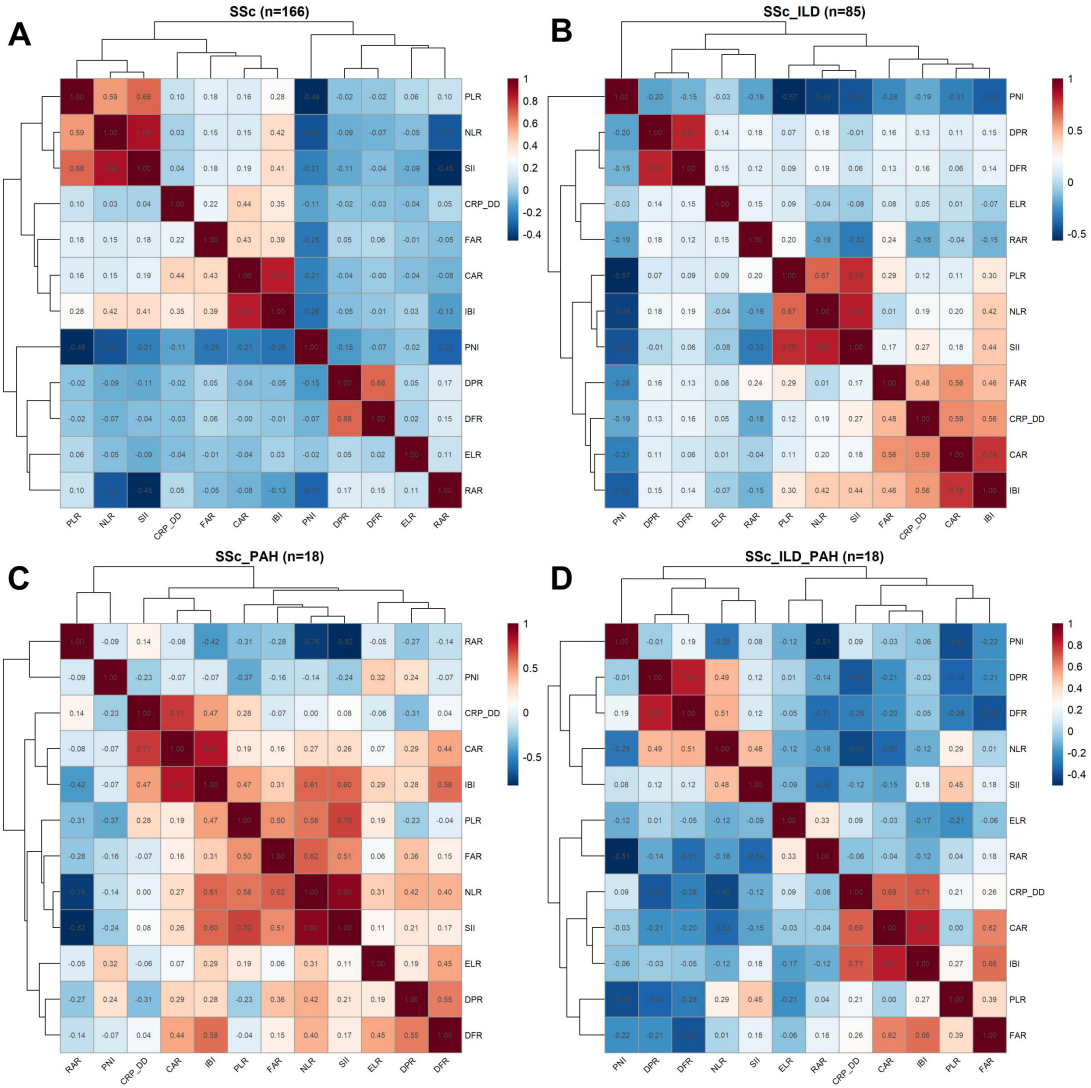
Spearman correlation heatmaps of 12 IICF indicators in different SSc subgroups. **(A)** SSc only group (n=166); **(B)** SSc-interstitial lung disease (SSc-ILD) group (n=85); **(C)** SSc-pulmonary hypertension (SSc-PH) group (n=18); **(D)** SSc-ILD-PH group (n=18). The color scale ranges from -1 (perfect negative correlation, dark blue) to 1 (perfect positive correlation, dark red).

In the correlation analysis of the 12 IICF markers, Spearman’s rank correlation coefficient was used to assess the strength of association between the markers. The results showed widespread moderate to strong correlations among the markers. Inflammatory and immune activation markers exhibited significant positive correlations, with SII showing the strongest correlation with NLR (r = 0.85), and IBI also demonstrating a very strong positive correlation with ELR (r = 0.82); Furthermore, PLR was highly positively correlated with both NLR (r = 0.60) and IBI (r=0.72). Nutritional indicators, on the other hand, were predominantly negatively correlated with markers of inflammation and immune activation. Specifically, the PNI showed a moderate negative correlation with the PLR (r = −0.48) and with the SII (r = −0.43), suggesting an inverse relationship between immune activation and nutritional status. There is also a certain association between some coagulation-related indicators and inflammatory indicators; for example, FAR and CAR show a moderate positive correlation (r = 0.47), whilst RAR and NLR (r = −0.31) and PNI and NLR (r = −0.39) exhibit a weak negative correlation. The network graph constructed using a threshold of |r| > 0.3 and P < 0.05 (**Figure 1B**) further delineated a modular association structure among the indices. Subgroup analyses revealed some variation in the magnitude of inter-index correlations across the four patient groups (**Figure 2**; **Supplementary Material 2**); however, the high correlations among SII, NLR, IBI, and PLR are largely driven by their shared neutrophil and lymphocyte components and therefore reflect mathematical collinearity rather than disease-specific biological network reorganization. The observed correlation patterns should be interpreted primarily as reflecting shifts in the underlying neutrophil-lymphocyte balance rather than evidence of distinct network topologies. Owing to the limited sample size of the SSc−PH group (n = 18), fewer edges satisfied the predefined threshold; nonetheless, CRP_DD displayed relatively robust associations with multiple indices within this subgroup: the correlation coefficient between CRP_DD and IBI was 0.71; and with CAR was 0.69 (both p < 0.05). Overall, a complex network of correlations exists between inflammatory, immune activation and nutritional metabolic markers, whilst the strong correlations among certain coagulation and fibrinolysis markers suggest that they may reflect specific pathophysiological pathways.

### Association of the 12 indices with prognosis and ROC analysis

**Figure 3:**
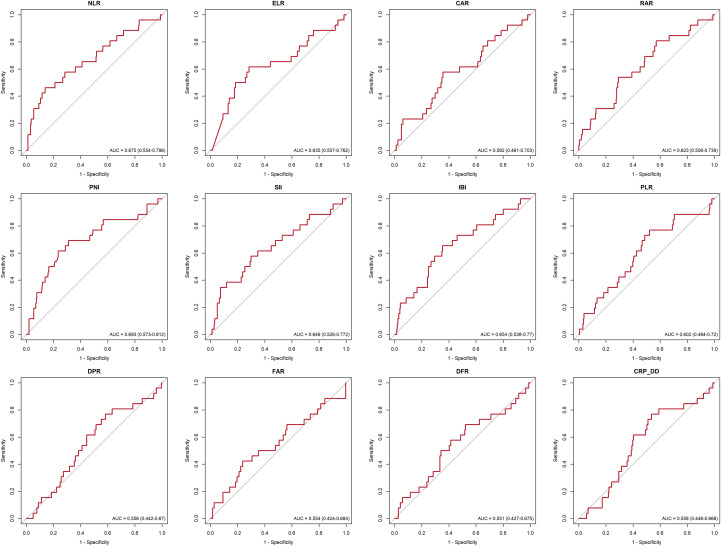
ROC curves of 12 single IICF indicators for predicting SSc mortality.

**Figure 4:**
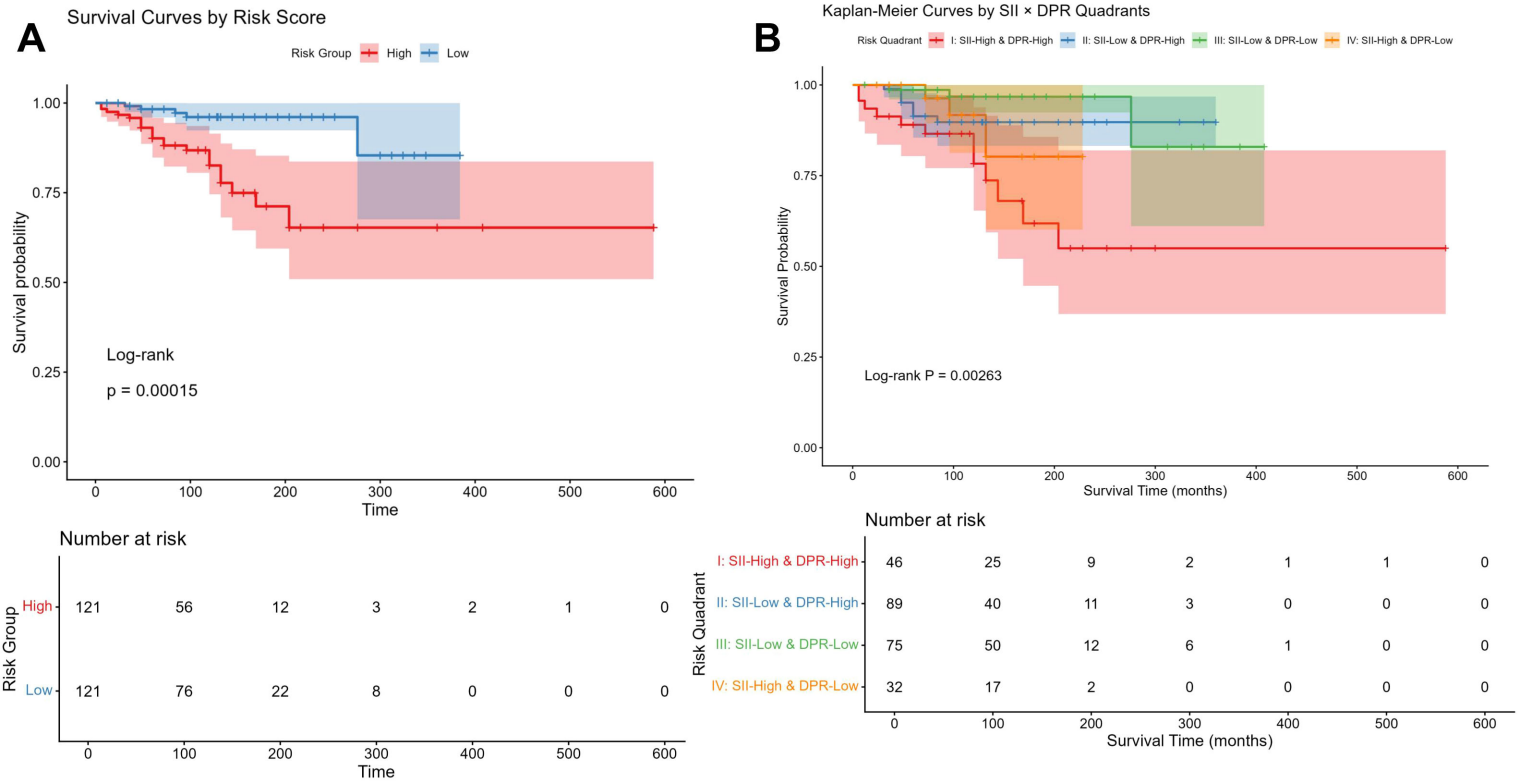
ROC curves of 12 single IICF indicators for predicting SSc mortality. **(A)** Kaplan-Meier survival curves for high-risk and low-risk groups stratified by the median of IICF-6 combined risk score. Survival differences were compared using the log-rank test. **(B)** Kaplan-Meier survival curves for four subgroups stratified by the optimal cut-off values of systemic immune-inflammation index (SII) and D-dimer to platelet ratio (DPR). Group I: High SII + High DPR; Group II Low SII + High DPR; Group III: Low SII + Low DPR (reference group); Group IV: High SII + Low DPR.

With patient prognosis designated as the outcome variable, Spearman correlation analysis was performed to examine the relationships between each of the 12 indices and prognosis (**Supplementary Material 3**). Inflammation−related indices, including CAR, NLR, and ELR, exhibited positive correlations with adverse prognosis, whereas PNI was inversely correlated with prognosis. Receiver operating characteristic (ROC) curve analysis (**Figure 3**) was subsequently conducted to evaluate the discriminatory capacity of each index for prognosis; the corresponding area under the curve (AUC) values, optimal cut−off thresholds, sensitivity, specificity, and Youden indices are summarized in the accompanying **Table 2** and **Supplementary Material 4**. The matrix of P values derived from DeLong tests indicated statistically significant differences in diagnostic performance between certain index pairs. The multi-index prognostic score yielded an area under the curve of 0.747 (95% CI 0.635–0.859) for mortality prediction. In pairwise DeLong comparisons, the combined score showed significantly higher AUC than CAR (P = 0.0148), RAR (P = 0.0055), SII (P = 0.0272), PLR (P = 0.0202), DPR (P = 0.0220), FAR (P = 0.0194), DFR (P = 0.0298), and CRP_DD (P = 0.0102), while differences with NLR (P = 0.1091), ELR (P = 0.0586), PNI (P = 0.3993), and IBI (P = 0.1055) did not reach statistical significance. Given the limited predictive power of univariate assessment, all 12 indices were subjected to LASSO−Cox regression for regularized variable selection. After excluding variables that resulted in unstable estimates due to complete separation, eight indices were retained in the final multivariable Cox proportional hazards model. Ultimately, only NLR (HR = 1.219, 95% CI: 1.003–1.483, P = 0.047) and DFR (HR = 2.188, 95% CI: 1.110–4.313, P = 0.024) emerged as independent risk factors for mortality in patients with SSc (**Supplementary Material 5**). The risk score derived from this model yielded an AUC of 0.747 (95% CI: 0.635–0.859) for predicting disease progression, representing an improvement over the individual indices (NLR alone AUC = 0.675; DFR alone AUC = 0.551) (**Supplementary Material 6**). Patients were dichotomized into high−risk and low−risk groups based on the median risk score, and Kaplan–Meier survival curves demonstrated a significant difference in survival between the two groups (log−rank test, P < 0.05), thereby corroborating the clinical utility of multidimensional IICF indices for prognostic stratification in SSc (**Figure 4A**).

**Table 2:** AUC, optimal cut-off value, sensitivity, specificity and the Youden index for the 12-variable model.

Indicator	AUC	CI-lower	CI-upper	Threshold	Sensitivity	Specificity	Youden
NLR	0.674	0.553	0.795	4.479	0.461	0.862	0.323
ELR	0.634	0.507	0.762	0.021	0.615	0.716	0.331
CAR	0.581	0.461	0.702	0.091	0.576	0.643	0.220
RAR	0.622	0.506	0.739	1.868	0.538	0.708	0.247
PNI	0.692	0.573	0.812	45.175	0.692	0.689	0.381
SII	0.648	0.526	0.771	748.144	0.576	0.701	0.278
IBI	0.654	0.538	0.770	11.074	0.653	0.647	0.301
PLR	0.602	0.484	0.720	135.868	0.769	0.478	0.248
DPR	0.556	0.442	0.669	0.001	0.769	0.417	0.186
FAR	0.554	0.424	0.684	0.098	0.423	0.762	0.185
DFR	0.551	0.427	0.675	0.165	0.692	0.478	0.171
CRP_DD	0.557	0.447	0.667	11.805	0.769	0.463	0.232

NLR, Neutrophil-to-lymphocyte ratio; ELR, Eosinophil-to-lymphocyte ratio; CAR, C-reactive protein-to-albumin ratio; RAR, Red cell distribution width-to-albumin ratio; PNI, Prognostic nutritional index; SII, Systemic immune-inflammation index; IBI, Inflammatory burden index; PLR, Platelet-to-lymphocyte ratio; DPR, D-dimer-to-platelet ratio; FAR, Fibrinogen-to-albumin ratio; DFR, D-dimer-to-fibrinogen ratio; CRP_DD, C-reactive protein-to-D-dimer ratio; AUC, Area under the receiver operating characteristic curve; CI, Confidence interval. The optimal cut-off value was determined by the maximum Youden index.

### Stratified analysis by immune–coagulation dual−axis

Patients with missing data for any of the 12 core indices, survival time, or outcome status were excluded from this analysis, resulting in the removal of 45 cases and a final analytic sample of 242 patients. Based on the optimal cut−off values derived from ROC analysis for the immune−axis representative index SII (748.14) and the coagulation−axis representative index DPR (0.0018), the 242 patients were classified into four quadrants: Quadrant I (high SII + high DPR, dual−elevation type), 46 cases (19.0%); Quadrant II (low SII + high DPR, coagulation−dominant type), 89 cases (36.8%); Quadrant III (low SII + low DPR, dual−low type), 75 cases (31.0%); and Quadrant IV (high SII + low DPR, immune−dominant type), 32 cases (13.2%). Kaplan–Meier survival curves revealed a statistically significant difference in survival among the four groups (log−rank P = 0.003). In Quadrant I there were 8 deaths among 46 patients (17.4%), in Quadrant II 6 deaths among 89 patients (6.7%), in Quadrant III 2 deaths among 75 patients (2.7%), and in Quadrant IV 1 death among 32 patients (3.1%). The total number of events was 17.With Quadrant III (dual−low type), which exhibited the most favorable prognosis, serving as the reference category, Cox regression analysis demonstrated that patients in Quadrant I (dual−elevation type) had a markedly increased risk of mortality (HR = 7.41, 95% CI: 2.09–26.30, P = 0.002). The HRs for Quadrant II (coagulation−dominant type) and Quadrant IV (immune−dominant type) were 2.77 (95% CI: 0.73–10.48, P = 0.133) and 3.06 (95% CI: 0.61–15.29, P = 0.173), respectively. Pairwise comparisons indicated that only the difference in survival between Quadrant I and Quadrant III reached statistical significance (P < 0.001), whereas the differences between Quadrants II and III (P = 0.137) and Quadrants IV and III (P = 0.091) were not statistically significant. These findings indicate that concomitant elevation of both SII and DPR confers the poorest prognosis, whereas dual−low expression is associated with the most favorable outcome. The immune–coagulation dual−axis stratification thus enables effective identification of high−risk SSc patient subsets. Notably, isolated coagulation or immune abnormalities did not independently confer a significantly increased mortality risk; rather, only the concurrent presence of both abnormalities was associated with heightened risk. See **Figure 4B** for details.

### Dimensionality reduction and unsupervised clustering based on the 12 indices

**Figure 5:**
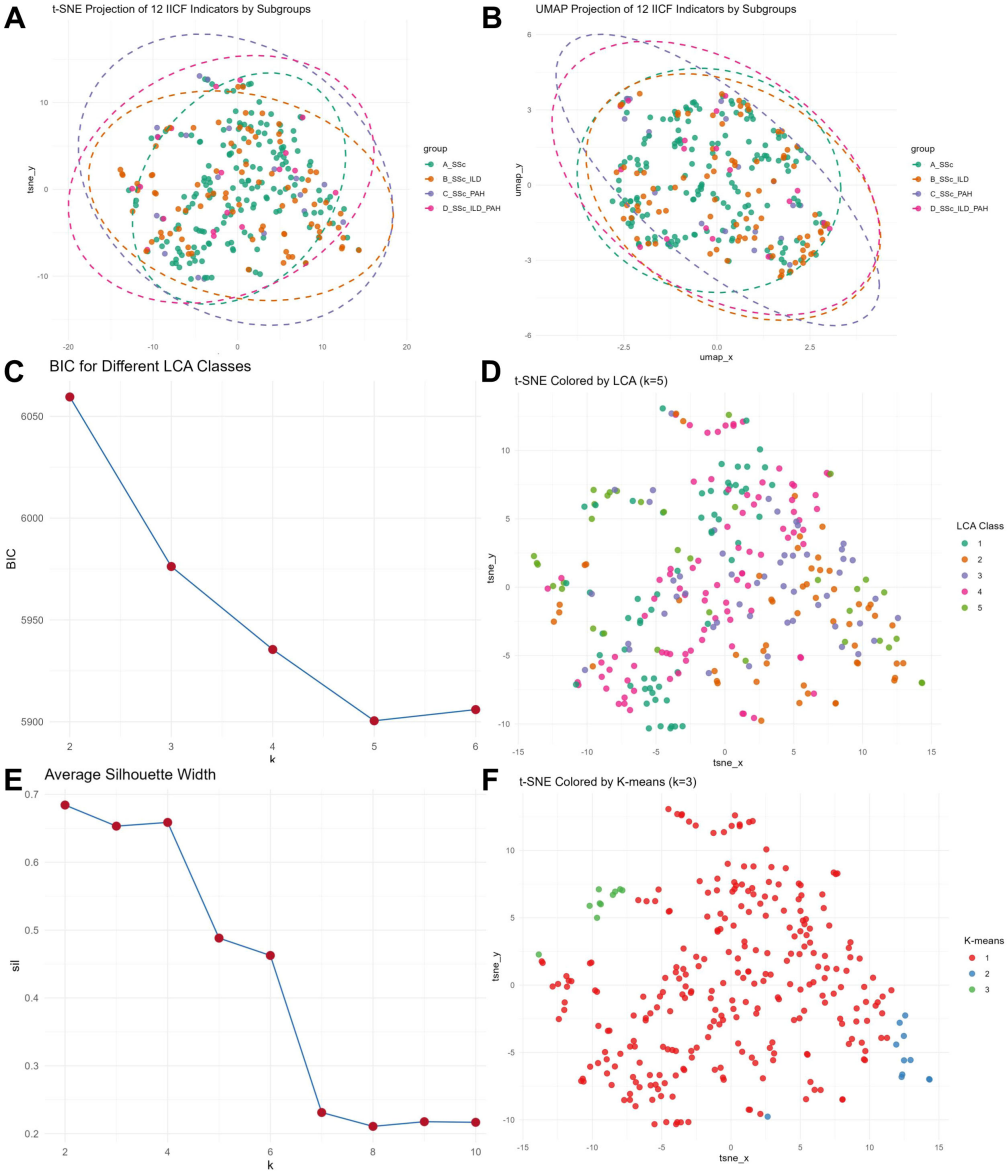
Dimensionality reduction visualization and clustering number determination. **(A)** t-distributed stochastic neighbor embedding (t-SNE) visualization colored by clinical subgroups. **(B)** Uniform manifold approximation and projection (UMAP) visualization colored by clinical subgroups. **(C)** Elbow method for determining the optimal number of K-means clusters (x-axis: number of clusters, y-axis: within-cluster sum of squares). **(D)** t-SNE visualization colored by latent class analysis (LCA) results. **(E)** Silhouette coefficient analysis for K-means clustering (x-axis: number of clusters, y-axis: average silhouette width). **(F)** UMAP visualization colored by LCA results.

To explore the potential utility of IICF indices for identifying distinct SSc patient subtypes, t−SNE and UMAP dimensionality reduction were performed following Z−score standardization of the 12 indices for all 287 patients (**Figures 5A, B**). Both methods revealed considerable overlap among the four clinical groups in the two−dimensional projection space, indicating that the four groups could not be clearly demarcated. Nonetheless, the SSc−ILD and SSc−ILD−PH groups exhibited a tendency to aggregate toward specific regions, suggesting that the presence of ILD may be accompanied by a relatively distinct IICF signature profile.

### Latent class analysis

To investigate potential latent disease subtypes based on IICF indices, the 12 continuous indices were discretized into ordered categorical variables according to tertile distributions, and latent class analysis (LCA) was performed. Models specifying 2 to 6 latent classes were fitted, and the optimal number of classes was determined by integrating the Bayesian information criterion (BIC), posterior class probabilities, and class sample size distribution. Although the BIC exhibited a marginal further decline at k = 5, the reduction in BIC from k = 5 to k = 6 was merely 0.09%, and the smallest class proportion in the 6−class model approached the 5% threshold. Considering both model parsimony and clinical interpretability, the 5−class model was ultimately selected as the optimal solution (**Figure 5C**). To visually assess the actual discriminatory capacity of the 5−class LCA classification, t−distributed stochastic neighbor embedding (t−SNE) was employed to project the 12−dimensional IICF data onto a two−dimensional plane. The visualization demonstrated that the five latent classes delineated by the LCA algorithm were almost entirely intermingled in the two−dimensional space, failing to form any distinct, well−separated clusters, with only weak local aggregation tendencies apparent. This finding indicates that, based on the 12 IICF indices included in the present study, the 5−class latent classification derived from LCA lacks substantive structural discrimination within the high−dimensional immune−coagulation feature space (**Figure 5D**).

### K−means clustering analysis

The optimal number of clusters for K−means clustering was determined jointly by the elbow method and the average silhouette coefficient. The elbow method plot revealed a continuous, smooth decline in the within−cluster sum of squares (WSS) as the number of clusters (k) increased, with no discernible “elbow” inflection point, thereby precluding definitive determination of the optimal k value based on this method alone (**Supplementary material 7****).**

**Figure 6:**
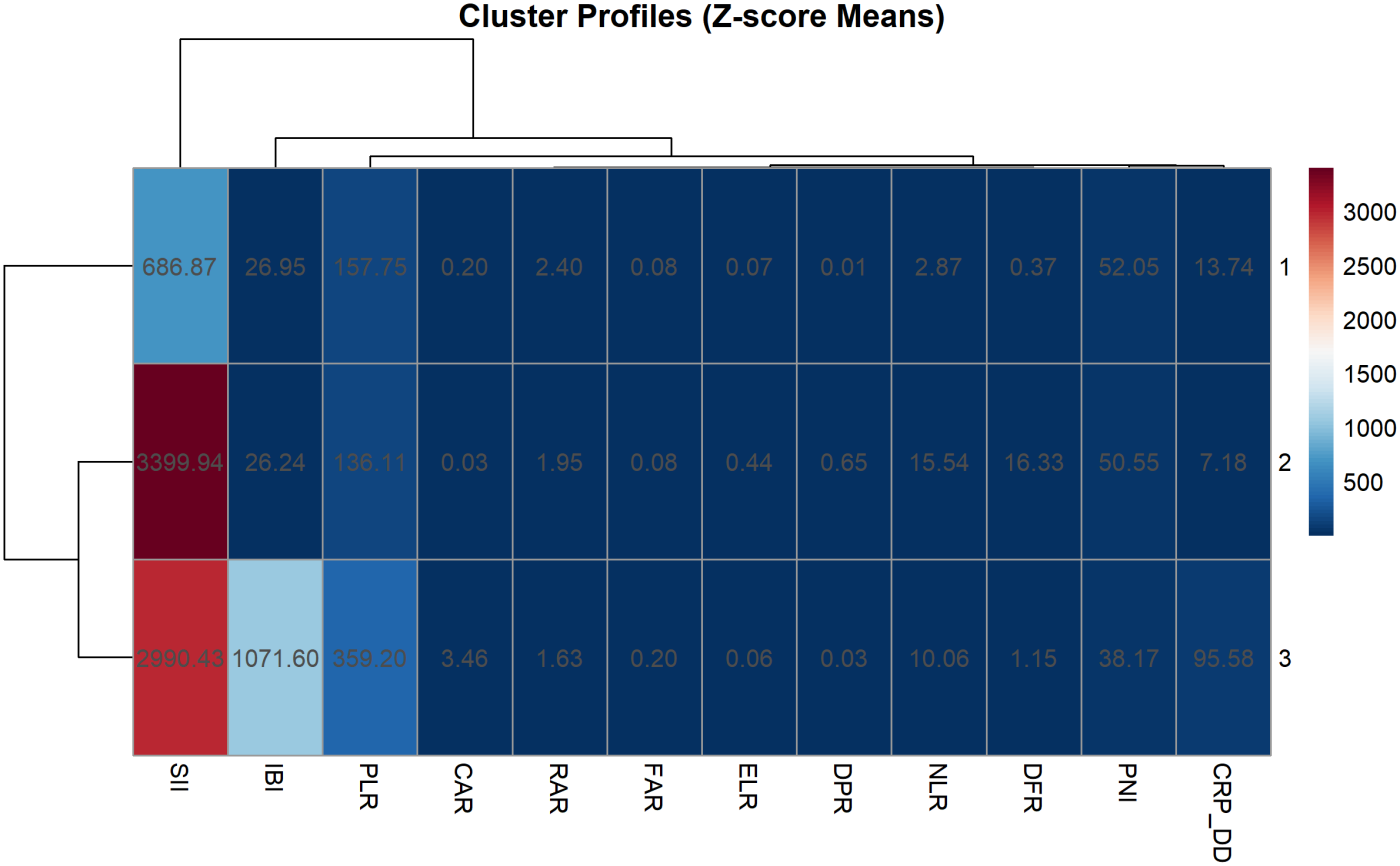
Z-score heatmap of 12 IICF indicators in three k-means clusters. Color intensity reflects original mean values (red high, blue low). Hierarchical clustering of variables and clusters was based on Euclidean distance and Ward’s method. Cluster 1: low inflammation, high nutritional index; Cluster 2: high inflammation with elevated coagulation/fibrinolysis markers; Cluster 3: high inflammatory indices. Their distribution was not fully concordant with clinical subtypes, underscoring heterogeneity of the immune–coagulation network.

The average silhouette coefficient provided supplementary evidence for assessing clustering quality: the highest average silhouette coefficient (≈0.68) was attained at k = 2, indicating optimal clustering performance; at k = 3, the coefficient decreased slightly but remained at a relatively high level (≈0.66); for k ≥ 4, the average silhouette coefficient declined precipitously, stabilizing at approximately 0.2 for k ≥ 7, signifying a marked deterioration in clustering quality. Integrating both metrics, k = 3 was selected as the optimal number of clusters for K−means and was employed for subsequent visualization (**Figure 5E**). Although the highest mean silhouette coefficient was observed at k=2, the two cluster solution grouped patients with elevated inflammatory responses yet heterogeneous coagulation profiles into a single cluster, thereby masking the immune–coagulation interplay that is central to our hypothesis. The k=3 solution, despite yielding a slightly lower silhouette coefficient, partitioned patients into biologically interpretable phenotypes: a low-inflammation, well-nourished phenotype; a hyperinflammatory, hypercoagulable phenotype; and an isolated hyperinflammatory phenotype, thus aligning with the conceptual framework of IICF network imbalance proposed in this study. K−means clustering partitioned the patients into three IICF phenotypic clusters (**Figure 5F**). Cluster 1 was characterized by low inflammation and a high nutritional index; Cluster 2 exhibited high inflammation coupled with elevated coagulation/fibrinolysis markers; and Cluster 3 was defined by high inflammatory indices (**Figure 6**). The distribution of these clusters was not fully concordant with the clinical ILD/PH grouping, underscoring the existence of immune–coagulation network heterogeneity that transcends conventional clinical stratification.

## Discussion

This study systematically constructs a 12-index network encompassing immune, inflammatory, coagulation, and fibrinolytic pathways in systemic sclerosis and an exploration of its topological features across distinct organ involvement phenotypes. Our principal findings are threefold. First, immune-coagulation dual-axis stratification based on the Systemic Immune-Inflammation Index and D-dimer to Platelet Ratio revealed distinct prognostic risks. Specifically, the concurrent elevation of both indices conferred a 7.41-fold increased risk of mortality compared to the dual-low phenotype, whereas elevation of a single axis did not independently reach statistical significance (HR 7.41, 95% CI 2.09-26.30, P = 0.002). Second, K-means clustering identified distinct molecular endotypes characterized by low inflammation with preserved nutrition, high inflammation with elevated coagulation markers, and isolated high inflammation. These endotypes showed incomplete concordance with clinical ILD and PH subgroups, indicating that the IICF signature transcends conventional organ-based classification. Third, a combined multi-index risk score derived via LASSO-Cox regression exhibited superior predictive performance for mortality with an area under the curve of 0.747, outperforming individual markers such as the PNI at 0.693 and the NLR at 0.675. Fourth, correlation network topology differed across organ-involvement phenotypes: patients with ILD or combined ILD-PH exhibited enhanced connectivity between coagulation/fibrinolysis and immune activation modules, whereas isolated SSc was characterized by dense intra-inflammatory clustering. Collectively, these findings suggest that adverse outcomes in systemic sclerosis are driven not by isolated pathway aberrations but rather by a systemic disequilibrium of the immune-inflammatory-coagulation-fibrinolysis network, with particular emphasis on the synergistic interplay between immune activation and coagulation dysregulation. The incomplete concordance between IICF molecular endotypes and clinical ILD/PH subgroups merits careful interpretation. From a clinical perspective, some patients with isolated SSc were assigned to the high-inflammation or hypercoagulable molecular subtypes despite lacking imaging or hemodynamic evidence of ILD or PH. These patients may represent a subclinical phase of network dysregulation in which systemic immune-coagulation imbalance precedes overt organ manifestations, and their long-term risk of organ progression may be underestimated by conventional assessment. From a methodological perspective, clinical ILD/PH classification relies on organ-level structural or hemodynamic thresholds that may not directly reflect the upstream systemic network state. Conversely, the IICF indices capture circulating biomarkers of network activity that can be abnormal before or independently of organ-level diagnostic criteria. This dissociation underscores the complementary nature of molecular phenotyping alongside conventional organ-based classification.

The core findings of this study substantiate that network imbalance, rather than isolated biomarker abnormalities, constitutes a critical driver of systemic sclerosis progression. While the SII and DPR each showed limited independent prognostic value in the overall cohort, their interaction proved pivotal as only their concurrent elevation was associated with a dramatically heightened mortality risk. This phenomenon may be mechanistically attributed to the excessive formation of neutrophil extracellular traps (NETs), which simultaneously activate innate immunity and provide a scaffold for platelet adhesion and coagulation cascade amplification, culminating in microthrombosis and secondary fibrinolysis (24). Kevin Didier et al. have found that, Polymorphonuclear neutrophils (PMN) from SSc patients were significantly more prone to releasing NETs than control PMN after autologous stimulation (25). PMN from patients with severe vascular complications (pulmonary arterial hypertension, digital ulcers) produced more NETs than PMN from other SSc patients and their aberrant NET production appeared to be sustained over time (25)In this context, SII captures NET-associated neutrophil–platelet activation, whereas DPR reflects fibrinolytic turnover secondary to NET-driven microthrombosis, explaining why their joint elevation—rather than either alone—marks the most severe network dysregulation. Besides, mitochondrial DNA-driven (mtDNA) auto-amplification loops further entrench the coupling between immune activation and coagulation (26). Research found that median plasma mtDNA levels were 152-fold higher in patients with SSc compared with healthy controls, whereas nuclear DNA levels were similar. SSc-derived mtDNA, in turn, acts as a potent DAMP that amplifies NETosis, particularly in SSc neutrophils, and triggers platelet activation, thereby establishing a self-sustaining release loop (27). However, the role of platelets in the pathogenic mechanism does not end there. Platelets represent a source of HMGB1 in the vasculature of SSc patients, possibly contributing to persistent microvascular injury and endothelial cell activation (28). Maugeri et al. report that platelet activation results in the translocation from the cytoplasm to the surface of HMGB1, a prototypical DAMP signal associated with tissue regeneration and the release of platelet-derived microparticles (PMPs) expressing HMGB1 (29). Platelet HMGB1 depletion is significantly associated in SSc patients with degranulation and with expression of P-selectin and of tissue factor as well as with fibrinogen binding to their plasma membrane (30). Collectively, these findings underscore that platelet activation bridges DAMP amplification and coagulation dysregulation, providing a mechanistic rationale for the immune–coagulation coupling observed in our IICF index profiling This study identified three IICF molecular subtypes using K-means clustering: the low-inflammation subtype with preserved nutritional status, the high-inflammation subtype with elevated coagulation markers, and the isolated high-inflammation subtype. This finding is of significant importance at both the clinical and mechanistic levels. From a clinical perspective, some patients with isolated systemic sclerosis have been classified into the high-inflammation or hypercoagulable molecular subtypes despite the absence of imaging or hemodynamic evidence of interstitial lung disease (ILD) or pulmonary hypertension (PH). Such patients may be in a compensatory or subclinical phase of network dysregulation, and their long-term risk of organ progression may be underestimated by conventional assessment methods. From a mechanistic perspective, this inconsistency suggests that although ILD and PH differ markedly in their end-organ manifestations, their upstream drivers may share a common basis of IICF network dysregulation. In patients with SSc-ILD and those with combined SSc-ILD-PH, network connectivity between coagulation and fibrinolysis markers and immune activation markers was enhanced, indicating that pulmonary involvement is accompanied by tighter immuno-coagulation interplay. Jaffar et al. found that blood-derived clotting factor XII is normally confined to the circulation but it leaks from damaged vessels into the lung interstitium in idiopathic pulmonary fibrosis where it induces IL-6 production and enhances migration of resident fibroblasts, critical events that drive chronic inflammation and therefore, contribute to fibrotic disease progression (31). Beyond its classic function in fibrin degradation, fibrinolysis also plays a broader role in regulating angiogenesis, vascular remodeling and tissue repair. Increased plasminogen activator inhibitor-1 (PAI-1) expression has been observed in the skin, lungs, and serum of patients with SSc (32, 33). In the vasculature, PAI-1 contributes to endothelial dysfunction by impairing fibrinolytic balance, inhibiting angiogenesis, and promoting vascular rarefaction (34, 35). In the context of fibrosis, PAI-1 inhibits ECM degradation and facilitates fibroblast activation, thereby contributing to tissue stiffening and scarring (36, 37). Consequently, the same fibrinolytic regulator promotes the development of pulmonary arterial hypertension in the pulmonary vasculature and the progression of fibrosis in the pulmonary stroma. This explains why an imbalance in the IICF network can manifest as ILD, PH, or both in different patients—with the specific phenotype depending on the relative dominance of key molecules such as PAI-1 in either the vascular or stromal compartments (38–41).

This study developed a multi-indicator combined risk score using LASSO-Cox regression to predict the risk of mortality. The prognostic nutritional index, which combines albumin and peripheral blood lymphocyte count, primarily reflects systemic metabolic wasting caused by chronic inflammation; the neutrophil-to-lymphocyte ratio primarily reflects the relative balance between innate and adaptive immunity; whilst the D-dimer-to-platelet ratio and the fibrinogen-to-albumin ratio reflect coagulation and fibrinolysis status. Individual markers can only capture information from a single node within the network, whereas the composite score integrates synchronous changes across multiple dimensions including inflammation, immunity, nutrition and coagulation, thereby mathematically better capturing the essential characteristics of systemic sclerosis as a network-based disease.

Several limitations of this study. First, the retrospective design precludes establishment of causality and introduces heterogeneity in follow-up duration and treatment regimens. The observed associations between network imbalance and prognosis require validation in prospective cohorts. Second, certain subgroups had limited sample sizes with only 18 patients each in the SSc-PH and SSc-ILD-PH groups. Furthermore, our subgroup definitions were based on established complications that themselves carry distinct prognostic weights, and this non-neutral stratification may confound the interpretation of inter-group network differences. For a retrospective cohort of this size, stratification by more neutral and biologically grounded criteria, such as autoantibody profiles (e.g., anti Scl70 versus anti centromere) or quantitative pulmonary function thresholds, would be preferable and should be adopted in future validation studies. These constraints may affect the stability of within subgroup correlation networks and statistical power, potentially limiting the generalizability of the findings. Third, pulmonary hypertension was defined by echocardiographic sPAP ≥36 mmHg without RHC confirmation in all cases. Given that a substantial proportion of SSc patients with elevated sPAP do not meet hemodynamic criteria for PH, the mortality signal attributed to these subgroups must be interpreted with caution and is more accurately understood as reflecting risk associated with echocardiographic evidence of elevated pulmonary pressure rather than confirmed PH. Future studies with routine RHC confirmation are essential to validate these findings. Fourth, inherent mathematical collinearity exists among several indices. Although ratio metrics partially mitigate the influence of absolute cell counts, indices such as the SII and PLR inherently share platelet and lymphocyte components. Their pathological independence requires further validation through experimental assays including flow cytometric detection of platelet activation markers. Fifth, inherent mathematical collinearity exists among several indices. Although ratio metrics partially mitigate the influence of absolute cell counts, indices such as SII, NLR, IBI, and PLR inherently share platelet, neutrophil, and lymphocyte components. Their high intercorrelations are algebraically expected under any condition altering the neutrophil-lymphocyte balance and should not be interpreted as evidence of disease-specific biological network remodeling. Future studies should employ orthogonal, mathematically independent assays to validate whether genuine network-level biological reorganization occurs. The wide confidence interval for the HR of 7.41 (95% CI 2.09–26.30) reflects the small number of events and that the point estimate should be interpreted with care pending validation in larger cohorts. Sixth, this is a single-center retrospective study and the IICF-6 risk score and dual-axis cut-off thresholds have not been externally validated. The generalizability of our findings to other populations, ethnicities, and healthcare settings remains uncertain. Future research should prioritize prospective multi-center cohort studies with standardized biomarker collection protocols and external validation in independent SSc cohorts to confirm the robustness and clinical utility of IICF-based stratification before its adoption in routine practice. Finally, the analysis was limited to routine clinical laboratory data and did not integrate novel inflammatory or fibrotic cytokines such as interleukin-6, calprotectin, or growth differentiation factor 15. Future integration of multi-omics data may further enhance model precision and biological insight.

Building upon these findings, future research should prioritize three directions. First, longitudinal dynamic monitoring of the IICF network is warranted to determine whether therapeutic interventions can induce favorable topological remodeling and whether a transition from the high-risk to the low-risk quadrant can serve as an early surrogate marker of treatment response; this requires prospective validation. Second, stratified interventional trials are needed to compare the safety and efficacy of standard immunosuppression plus anticoagulation versus immunosuppression alone in the dual-elevation high-risk subgroup, thereby validating the clinical utility of IICF-guided phenotypic stratification. Third, multi-omics integration frameworks that incorporate routine IICF laboratory metrics with genomic and proteomic profiling should be developed to elucidate the upstream molecular drivers of network dysregulation and to identify druggable therapeutic targets.

## Conclusion

In conclusion, by integrating a multidimensional network encompassing immune, inflammatory, coagulation, and fibrinolytic (IICF) pathways, this study demonstrates that systemic sclerosis harbors distinct immune-coagulation dual-axis imbalance endotypes that extend beyond conventional organ-based stratification. Notably, the synergistic interplay between immune activation and dysregulated coagulation/fibrinolysis constitutes an independent risk factor for markedly adverse outcomes. These findings underscore the clinical imperative of concurrent surveillance of inflammatory and coagulation parameters and suggest that patients exhibiting the dual-elevation profile may derive particular benefit from combined therapeutic interventions.

## Data Availability

The datasets used and analyzed during the current study are available from the corresponding author on reasonable request.
