# Supplementary material for: Immunological coagulation dual axis stratification identifies ultra high-risk phenotypes in systemic sclerosis
Source: Front Immunol. 2026 Jul 2;17:1866374. doi: 10.3389/fimmu.2026.1866374 (PMC13372969; doi:10.3389/fimmu.2026.1866374)
Supplement: Supplementary file 1 [file DataSheet1.docx]

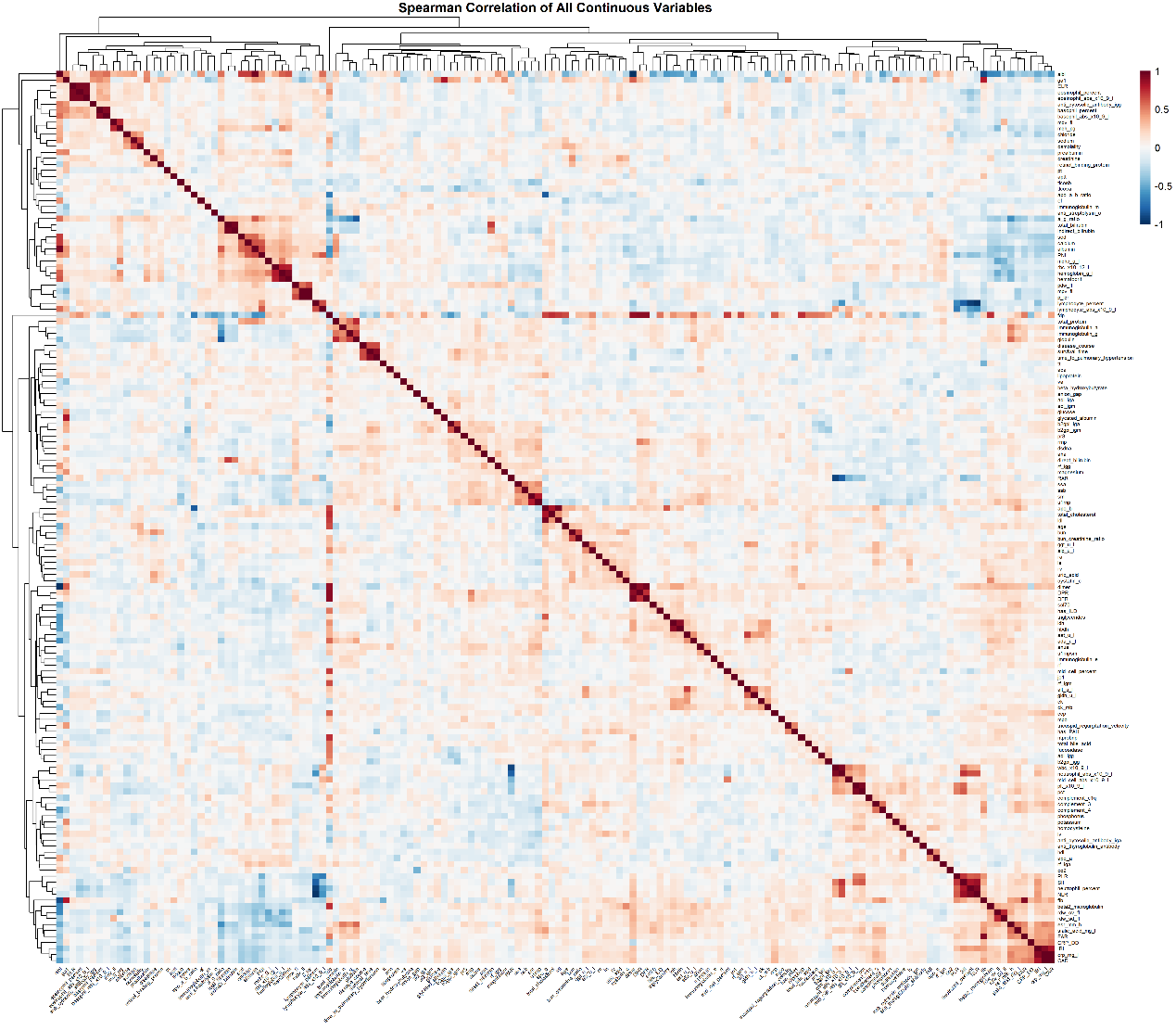


**Supplementary material 1**: Spearman Correlation Heatmap of All Continuous Variables.


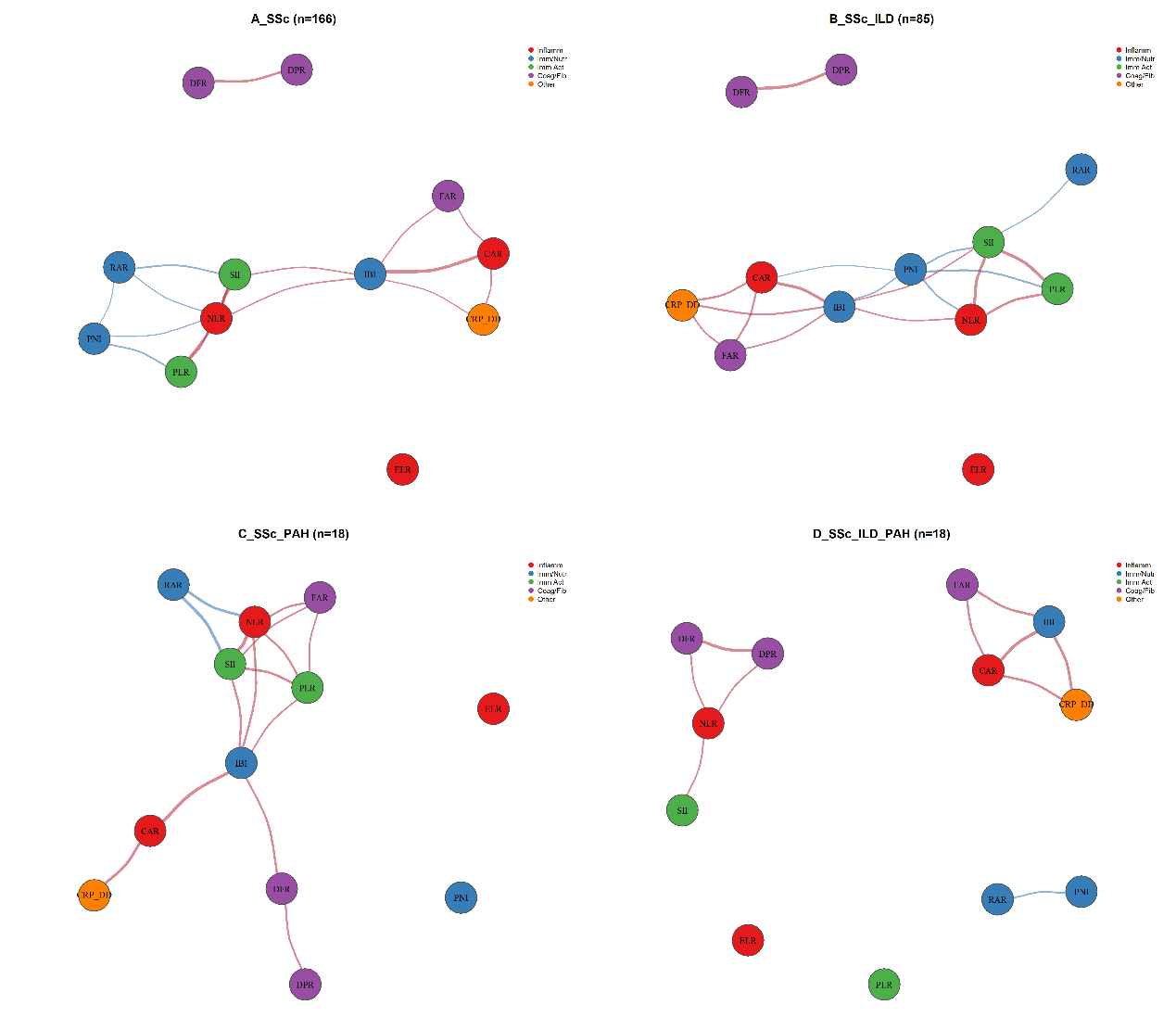


**Supplementary material 2.** Network analysis of IICF in systemic sclerosis across different clinical phenotypes.


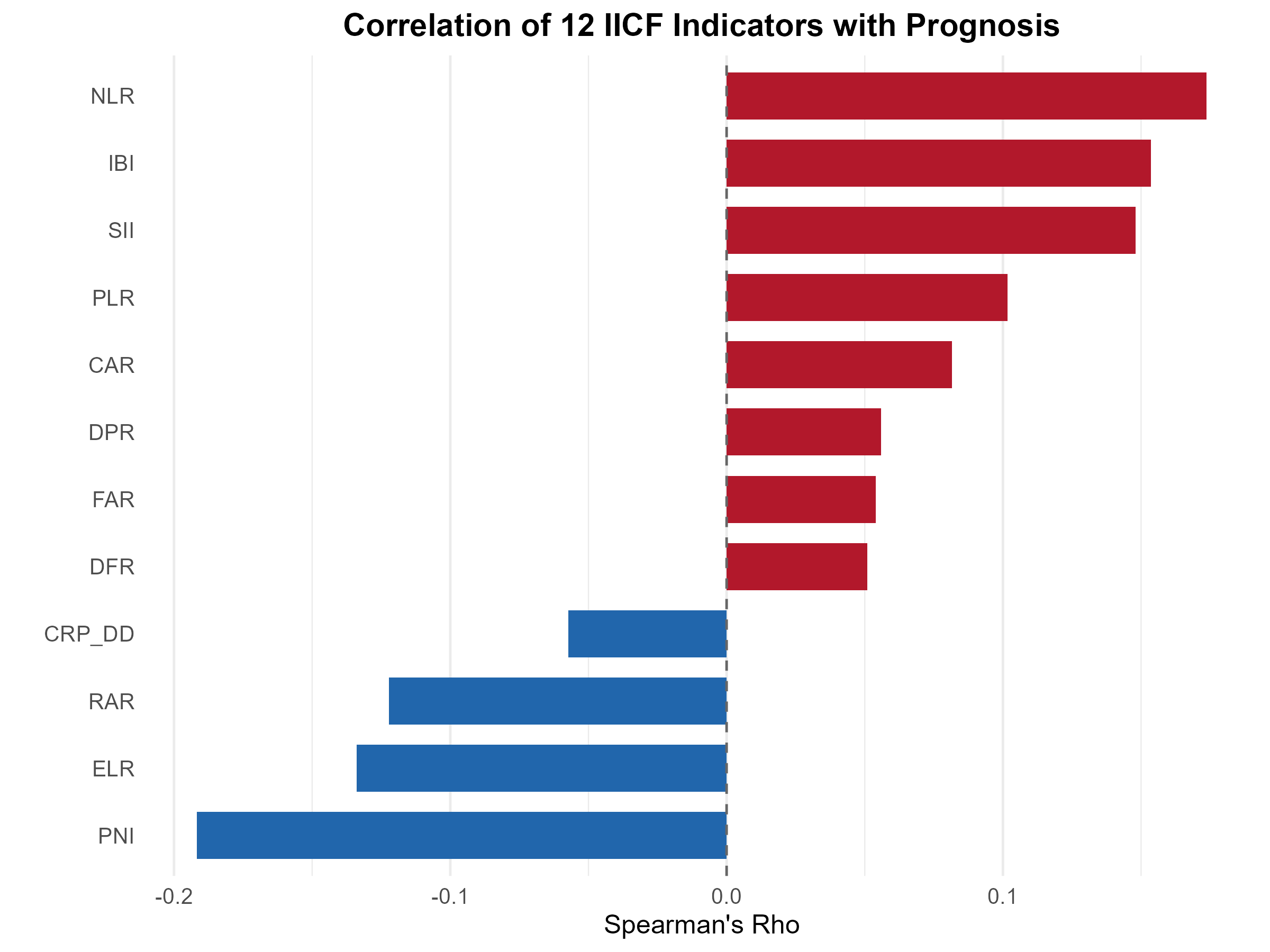


**Supplementary material 3**. Correlation Between 12 IICF Indicators and SSc Prognosis. Forest plot showing the Spearman correlation coefficients between each IICF indicator and all-cause mortality. Positive coefficients indicate a positive association with poor prognosis, while negative coefficients indicate a protective association.

**Supplementary material 4** P-value matrix for the DeLong test across various indicators.

|  | NLR | ELR | CAR | RAR | PNI | SII | IBI | PLR | DPR | FAR | DFR | CRP_DD |
| --- | --- | --- | --- | --- | --- | --- | --- | --- | --- | --- | --- | --- |
| NLR | - | 0.630 | 0.235 | 0.497 | 0.731 | 0.509 | 0.714 | 0.193 | 0.095 | 0.206 | 0.118 | 0.172 |
| ELR | 0.630 | - | 0.577 | 0.865 | 0.504 | 0.867 | 0.817 | 0.724 | 0.350 | 0.382 | 0.366 | 0.376 |
| CAR | 0.235 | 0.577 | - | 0.618 | 0.119 | 0.365 | 0.094 | 0.798 | 0.734 | 0.682 | 0.631 | 0.818 |
| RAR | 0.497 | 0.865 | 0.618 | - | 0.430 | 0.713 | 0.672 | 0.813 | 0.454 | 0.435 | 0.457 | 0.486 |
| PNI | 0.731 | 0.504 | 0.119 | 0.430 | - | 0.409 | 0.484 | 0.033 | 0.049 | 0.086 | 0.074 | 0.140 |
| SII | 0.509 | 0.867 | 0.365 | 0.713 | 0.409 | - | 0.914 | 0.350 | 0.251 | 0.276 | 0.262 | 0.347 |
| IBI | 0.719 | 0.817 | 0.094 | 0.672 | 0.484 | 0.914 | - | 0.384 | 0.168 | 0.216 | 0.149 | 0.335 |
| PLR | 0.193 | 0.724 | 0.798 | 0.813 | 0.033 | 0.350 | 0.384 | - | 0.593 | 0.560 | 0.588 | 0.635 |
| DPR | 0.095 | 0.350 | 0.734 | 0.454 | 0.049 | 0.251 | 0.168 | 0.593 | - | 0.982 | 0.900 | 0.982 |
| FAR | 0.206 | 0.382 | 0.682 | 0.435 | 0.086 | 0.276 | 0.216 | 0.560 | 0.982 | - | 0.970 | 0.973 |
| DFR | 0.118 | 0.366 | 0.631 | 0.457 | 0.074 | 0.262 | 0.149 | 0.588 | 0.900 | 0.970 | - | 0.935 |
| CRP_DD | 0.172 | 0.376 | 0.818 | 0.486 | 0.140 | 0.347 | 0.335 | 0.635 | 0.982 | 0.973 | 0.935 | - |

NLR: Neutrophil-to-lymphocyte ratio; ELR: Eosinophil-to-lymphocyte ratio; CAR: C-reactive protein-to-albumin ratio; RAR: Red cell distribution width-to-albumin ratio; PNI: Prognostic nutritional index; SII: Systemic immune-inflammation index; IBI: Inflammatory burden index; PLR: Platelet-to-lymphocyte ratio; DPR: D-dimer-to-platelet ratio; FAR: Fibrinogen-to-albumin ratio; DFR: D-dimer-to-fibrinogen ratio; CRP_DD: C-reactive protein-to-D-dimer ratio.

**Supplementary material 5**. Cox regression model.

| Variable | HR | Lower | Upper | P |
| --- | --- | --- | --- | --- |
| DFR | 2.188 | 1.110 | 4.313 | 0.024* |
| PLR | 1.007 | 0.998 | 1.016 | 0.111 |
| IBI | 1.003 | 0.997 | 1.008 | 0.351 |
| SII | 0.999 | 0.997 | 1.000 | 0.103 |
| PNI | 0.989 | 0.888 | 1.101 | 0.837 |
| CAR | 1.350 | 0.445 | 4.095 | 0.596 |
| NLR | 1.219 | 1.003 | 1.483 | 0.047* |
| ELR | 0.000 | 0.000 | 2.550 | 0.074 |

DFR: D-dimer-to-fibrinogen ratio; PLR: Platelet-to-lymphocyte ratio; IBI: Inflammatory burden index; SII: Systemic immune-inflammation index; PNI: Prognostic nutritional index; CAR: C-reactive protein-to-albumin ratio; NLR: Neutrophil-to-lymphocyte ratio; ELR: Eosinophil-to-lymphocyte ratio.HR: Hazard ratio; CI: Confidence interval. Variables included in the model were screened by LASSO-Cox regression with 10-fold cross-validation. The proportional hazards assumption was satisfied for all variables.


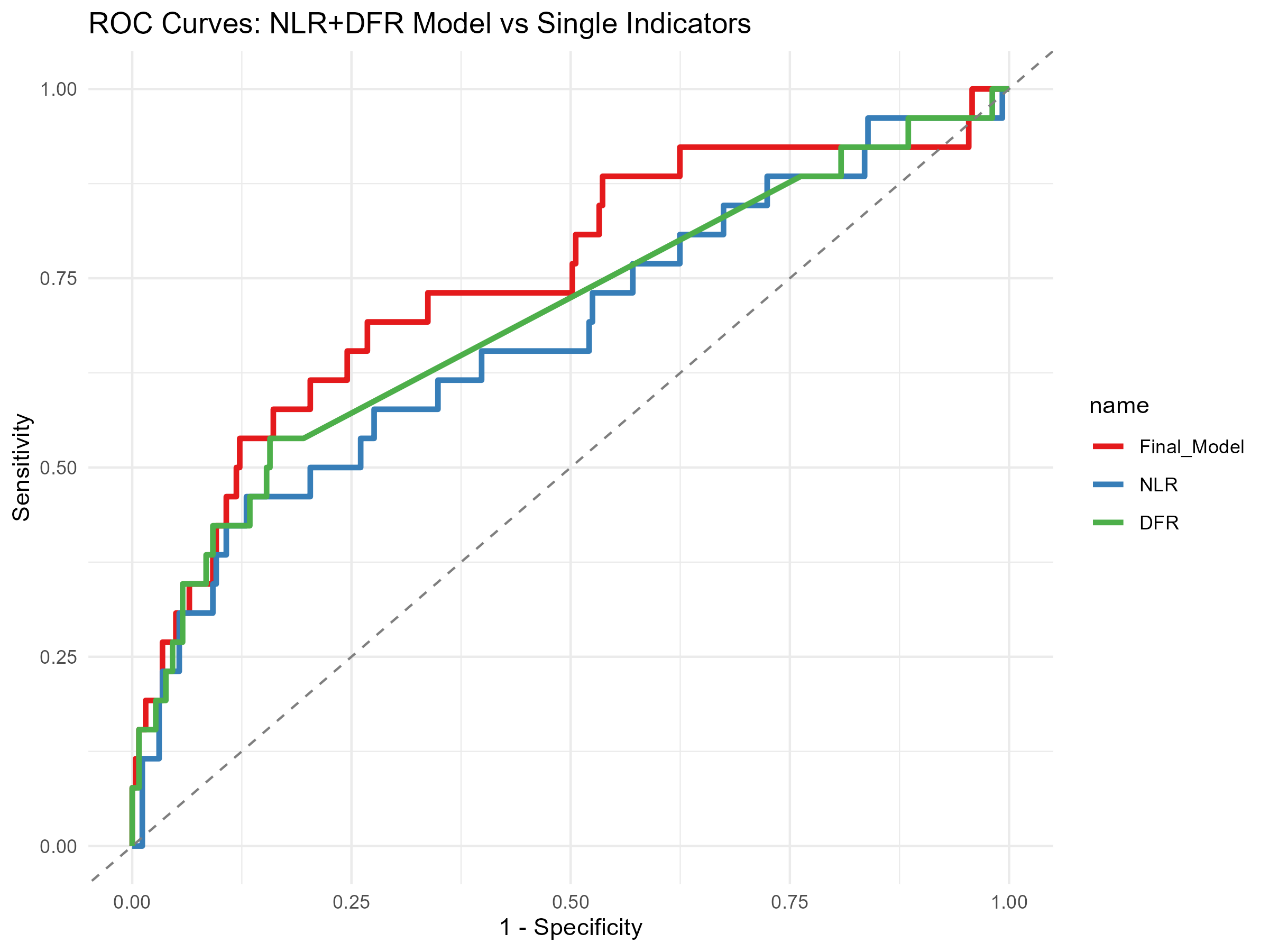


**Supplementary material 6.** Receiver operating characteristic curves comparing the diagnostic performance of the final model versus individual indicators.


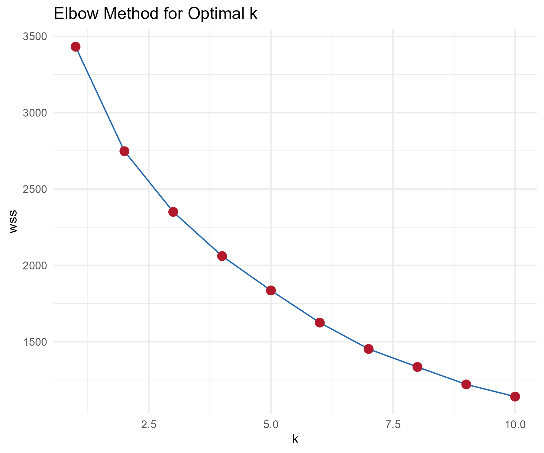


**Supplementary material 7.** Determination of the optimal number of clusters using the elbow method.
